# Tobacco industry accountability for marine pollution: country and global estimates

**DOI:** 10.1136/tc-2022-057795

**Published:** 2023-11-28

**Authors:** Deborah K Sy

**Affiliations:** 1 Global Public Policy & Strategy, Global Center for Good Governance in Tobacco Control (GGTC), Bangkok, Thailand; 2 Global Public Policy, HealthJustice, Manila, Philippines

**Keywords:** tobacco industry, environment, global health, low/middle income country, public policy

## Abstract

**Background:**

Commercial cigarette filters are single-use plastics and the main component of cigarette butts, the most common trash item collected worldwide. Governments bear the economic burden of managing the waste and the environmental pollution due to discarded filters and packages. Using available data sources, we estimate the economic burden of plastic tobacco waste on country economic groups.

**Methods:**

We reviewed available public data sources that could inform estimates of the economic environmental burden of butt waste for countries. We estimated total weight of plastic cigarette filters and packaging based on cigarette consumption and applied World Bank waste management cost estimates per ton to this total. We then applied estimates of ecosystem losses per ton of plastic waste provided by the World Wildlife Fund to establish losses attributable to tobacco’s plastics.

**Results:**

We estimate that US$25.7 billion is lost annually (waste management and marine ecosystem service losses) due to cigarette plastic sources. We estimate US$186 billion in such losses over a 10-year period, adjusted for inflation. Countries are making progress in developing plastics policies, particularly banning single-use ones, but the costs of tobacco’s plastic pollution are overlooked.

**Conclusion:**

Efforts to reduce plastic pollution should address cigarette filters as toxic, widespread and preventable sources of marine pollution. Countries may develop specific estimates of waste management and ecosystem costs in order to assign tobacco industry accountability for this pollution. These results indicate minimum estimates for a majority of countries.

**WHAT IS ALREADY KNOWN ON THIS TOPIC**

- The economic burden of tobacco’s toxic waste on a global scale is still a largely underexplored area, with most studies focusing on either a single kind of waste product (eg, cigarette filters) or a particular geographical location. The lack of waste management data specific to tobacco waste is a major limitation of such studies.

**WHAT THIS STUDY ADDS**

- This study highlights the concerns around tobacco’s toxic products as sources of single-use plastics, which are significant environmental contaminants, calculated for 194 countries, a first of its kind.

**HOW THIS STUDY MIGHT AFFECT RESEARCH, PRACTICE OR POLICY**

- The results of this study offer a practical and potentially useful method to estimate the economic burdens of tobacco plastics to countries according to their economic status, which can be further compensated by tobacco companies in the form of ecotaxes or penalties.

## Introduction

 Cigarette butts (or filters) are the most littered item on the planet, and numerous studies have demonstrated how they adversely affect aquatic and terrestrial life. These reports describe impacts on microorganisms, fish, amphibians, invertebrates, crustaceans and other life forms.^1^ Decades ago, cigarette filters were introduced under the guise of addressing health concerns related to smoking. However, the cellulose acetate filter, attached to nearly all commercial cigarettes, is a deceptive design element that makes cigarette smoking more appealing, especially to adolescents and young adults, by providing a false impression of safety.^2^ In actuality, cigarette filters have failed to mitigate the health risks associated with tobacco use for the general public.^3^ The article “Cigarette with Defective Filters Marketed for Over 40 Years: What Philip Morris Never Told Smokers” pointed out that the cellulose acetate fibers comprising the filter have been shown to deposit into the lungs of smokers.

In response to increasing public awareness about the environmental impacts of tobacco product waste, the tobacco industry (TI) has made attempts to ‘greenwash’ its activities. For example, the industry has engaged with anti-littering campaigns and coastal clean-up events to address these concerns through governmental and non-governmental activities.^4^,^7^ These include collection and recycling schemes,^8^,^10^ ashtray giveaways^11^ and water stewardship initiatives.^12^,^14^ Although these efforts try to put the TI in a good light, such downstream-focused remedies do not result in significant changes in the volume of tobacco product waste.^15^ Currently, around 70 countries ban all forms of such TI sponsorship (better known as so-called corporate social responsibility) under obligations of the WHO’s Framework Convention on Tobacco Control (FCTC).^16^

Governments bear the main burden of the direct and secondary environmental costs (externalities) of tobacco product waste, especially plastic cigarette filters.^17^ Thus, tobacco control and environmental agencies could benefit from estimates of the environmental costs due to tobacco plastics. There have been a handful of efforts to quantify these costs and assign responsibility for them to the industry (table 1). In most cases, the research has involved observational studies and municipal/national level estimates of clean-up costs. Data for these types of studies are lacking for the majority of countries. We describe an economic estimation process involving currently available data that can provide a minimum quantification of the environmental costs attributable to tobacco product plastics.

**Table 1:** Governmental efforts to hold tobacco industry accountable for tobacco product waste costs

Country	Cost amount/year
France	Government estimated *€80 million* annual cost of reducing environmental pollution caused by cigarette butts.^34^
UK	Government estimated *£40 million* annual cost of cleaning up discarded cigarette butts.^35^
San Francisco, California (USA)	Tobacco product waste clean-up cost in San Francisco alone is approximately *US$7.4 million* annually.^36^
European Union	No specific cost estimate is available for tobacco product waste, but ‘Single-Use Plastics Directive’ is estimated to reduce the environmental damage of these plastics by *€22 billion*.^37^

## Methods

We first reviewed existing sources and literature on tobacco’s environmental economic costs through a keyword search for ‘cost’, ‘valuation’, ‘estimate’, ‘cigarette butts’, ‘cigarette filters’, ‘plastic pollution’, ‘waste’, ‘current’ and ‘projected’. We found that there were estimates available from the World Bank’s publication, *What a Waste 2.0*, for waste management costs per volume collected in each country as well as efficiency costs (1-estimated percentage of waste not collected).^18^ ‘Benchmark’ costs for total plastic waste are available from the Organisation for Economic Co-operation and Development (OECD) report on plastics.^19^

Data on cigarette sales (consumption) are published periodically in the *Tobacco Atlas* at the country level.^20^ Not all cigarettes sold have filters; therefore, we used a schedule of 49 countries that lists a specific percentage of cigarettes that have filters and then assumed that 98% of cigarettes sold in the remainder of the countries are filtered.^21^ The average weight of 20 plastic filters is 3.4 g.^22 23^

Because cigarette butts are often littered along with plastic packaging, we included the weight of plastic packaging in the estimation procedure, which includes the outer plastic film as well as the inner sleeve. The weight of the package and sleeve is computed based on the standard size of a pack of 20 cigarettes (19 g).^24^

World Wildlife Fund (WWF) provided a range of economic estimates for marine ecosystem losses (MEL) that can result from a ton of plastic waste (US$204 270–US$408 541).^25^ We used the median (US$306 405.5) of this range to apply conservatively to all country estimates per ton of tobacco plastic waste.

Finally, we developed a formula to estimate the single-year and 10-year projections of the environmental economic costs of tobacco plastic based on the tonnage of cigarette filters and plastic packaging. We multiplied this quantity by MEL per ton and by the waste management costs per ton for countries according to World Bank economic grouping. This total reflects cost estimates of clean-up and disposal of the total plastic generated by filtered cigarette sales, and not the clean-up costs that might be estimated for tobacco waste that remains deposited in the environment. This estimate represents the potential costs of tobacco plastics that will end up as waste, either in our oceans, landfills or in the environment. Costs were adjusted for inflation for a 10-year period, which has been reported as the survival time for cigarette filters under varying environmental conditions.^26^

## Results

The general formula used for estimating the environmental economic costs of cigarette plastic pollution is:

Environmental Economic Cost of Cigarette Plastics (US$/year)=(EPW×TWMC/year)+(EPW×MEL/year)

where Estimated Plastic Waste (EPW) in tons=weight of filters+plastic packaging (from number of cigarettes consumed×filter weight in grams/stick+weight of plastic packaging for number of packs of 20 cigarettes×plastic required per pack and sleeves [metre of film]×plastic density [grams/metre]) and where *current* Tobacco Waste Management Costs (TWMC)/year=EPW (in tons)×Collection Efficiency (in %)×Waste Management Costs (WMC)/year (as reported by the World Bank), and where Marine Ecosystem Losses (MEL) are estimated, over a 10-year period.

$$Ten-year\;projected\;TWMC/year=\;EPW\;(intons)\times Uncollected\;Waste\;(in\%)\times \;‘Benchmark^{\prime }\;plastic\;waste\;cost/year\;(according\;to\;OECD\;estimates).$$

*Uncollected Waste=1-Collection Efficiency in %

Therefore,

MEL per year (over a ten10−year lifetime)=EPW (in tons)×Leakage Rate×median MEL per year

*Leakage Rate: Leakage rate of plastics into the environment=the small percentage (1–14% depending on country) of plastic waste that is presumed to leak into the oceans.

Based on this methodology, around US$26 billion is the annual economic cost of potential cigarette product plastic waste, including US$20.7 billion in ecosystem losses and US$5 billion in waste management costs (as shown in table 2). Although this may appear to be a relatively small amount in comparison to the annual economic losses caused by tobacco, which total US$1.4 trillion per year, it is still a significant cost in terms of cigarette plastic waste management, especially considering that it only pertains to one kind of tobacco product. Countries with the highest number of cigarette butts are mostly low and middle-income countries. These are the same countries where the ‘leakage’ rate for plastics into the environment is likely higher.^27^ Hence, costs are highest in countries such as China, Indonesia, Japan, Bangladesh and the Philippines (tables2  3).

**Table 2:** Waste management costs by country income classification

Income class	Butts (in millionsof tons	Package plastic (in millions of tons)	World Bank data range per ton (70–328US$)^18^	Waste managementcost: current (US$ '000)	OECD datarange per ton (274–406 US$)^19^	Waste managementcost: additional (US$ '000)	Total waste managementcost (US$ '000)
High income	198,670	368,474	328	183,039	274.91	2,266	184,835
Upper middle income	419,869	952,317	140	184,805	274.91	31,615	216,421
Low middle income	158,328	333,988	110	33,407	506.18	95,471	128,878
Low income	20,984	37,793	70	1,964	506.18	15,544	17,508
Total	797,851	1,692, 572	–	403,217	–	144 897	547 644

OECDOrganisation for Economic Co-operation and Development

**Table 3:** Marine pollution and waste management costs by income classification (in million US$)

Income class	Waste management cost (US$,000)	Loss of ecosystem service lifetime (marine pollution) (US$,000)	Total(US$,000,000)
High income	184,835	5,233,430	5,418
Upper middle income	216,421	6,098,970	6,315
Low middle income	128,878	8,460,670	8,589
Low income	17,508	620,881	638
Global	547,644	20,413,960	21,253

*Plastic leakage is the potential amount of macroplastics and microplastics that are not kept in a circular loop or properly managed at their end of life, and thus leak into the environment.

## Discussion

Annual costs of plastic waste due to commercial filtered cigarettes are substantial (approximately US$26 billion). Ecosystem losses are significantly higher than waste management costs since these account for long-term impact.

The estimate for ecosystem losses is arguably conservative since it is the median, not the upper estimate, of the MEL figure provided by WWF that was used. Further, the data used to estimate TWMC are averages for collecting and processing general waste. Cigarette butts typically require special clean-up, handling and management processes due to their small size, toxicity and ubiquity.

Over 10 years, the loss of ecosystem value would be around US$186 billion, accounting for inflation. Although this amount is small compared with the annual economic losses from tobacco (US$1.4 trillion per year)^22^ and may appear insignificant compared with the 8 million deaths attributable to tobacco each year, these environmental costs should not be downplayed as these are accumulating and are preventable.

### Limitations

Our TWMC estimation method does not account for the toxic properties of cigarette butts that makes them more harmful than only plastic waste. As mentioned, the cost of picking up tobacco product waste is likely to be significantly higher than total waste management costs in high-income countries. Cigarette butts are small and widely littered, and so they are harder to collect.^15^ Finally, it is difficult to estimate the accrual of tobacco pollutants over the years. These would include persistent toxic chemicals, metals and microplastics.^1^ Cigarette filters have been polluting our oceans and land for at least five decades, and these trash items may have a carrier effect with the toxic chemicals leached from them. Human and ecosystem impacts of this toxic chemical accumulation are unknown.

A localised, observational research approach would be required to make a more accurate estimate of tobacco’s environmental costs. However, few countries have undertaken such studies, despite the growing evidence for the environmental harms of tobacco growing, manufacturing, use and disposal.^22^

The general estimates provided here could provide fiscal evidence of the need to mitigate tobacco plastic waste pollution. Optimal costing studies involve collecting data at the country level, which may not be feasible or practical in many developing nations due to lack of funding, data or capacity. Where such data or research are not available, cost estimates derived from public sources as the World Bank, WHO and OECD can provide useful preliminary information.

## Conclusion and recommendations

Numerous nations around the world have united in their efforts to combat the issue of plastic pollution, including through a new treaty negotiation process that began in March 2022 and will conclude by December 2024.^28^ Cigarette butts, being one of the most littered items in the world, should be specifically addressed by a global plastic waste policy. Initial planning discussions on the plastics treaty suggest that plastic producers will be expected to bear some or all of the costs of prevention and mitigation of plastic pollution. So far, these costs are imposed on industries in only high-income countries, and they include only waste management and litter abatement (see online supplemental tables). To prepare for more specific economic policy discussions, governments need valid estimates of waste management costs and ecosystem losses. These data will assist in assigning industry responsibility for these losses, including that of the TI. The estimates herein represent a minimum estimate for a majority of countries; and for tobacco products, there will be additional costs of handling the toxic chemicals accompanying the plastic waste from cigarettes.

Shifting costs of tobacco product waste upstream to tobacco companies is crucial but is only a stopgap measure in terms of the tobacco end game, which is to accomplish a tobacco-free future.^29 30^ According to many advocates, the better solution is to ban the sale of plastic cigarette filters as part of the global effort to eliminate single-use plastics.^2^ Cigarette butts are unlike any other single-use plastic waste product; filters have no beneficial use and are a marketing feature that only makes smoking more palatable. The TI knew filters were a design flaw in that their use has been linked to a specific type of aggressive lung cancer, in addition to other cancer and cardiovascular risks caused by smoking tobacco.^31^ It bears stressing that because the TI continues to market the most hazardous consumer product in the world, along with such a toxic product feature, and because of its history of human rights violations,^32^ it should be excluded from any intergovernmental engagements such as the plastics treaty. This rogue industry is not a ‘stakeholder’ in efforts to improve human health or environmental safety. The tobacco companies have significant control over the product design and supply chains of their products, and the entire life cycle of tobacco production and use causes harms to the environment.^22^ They have chosen to obscure and aggravate these harms through new products (eg, electronic cigarettes) that create additional sources of environmental pollution.^33^

Cost estimates must also include accrued harms. This approach aligns with environmental principles such as ‘Polluter Pays Principle’, the application of which would be consistent with the governments’ duty to deal with tobacco industry liability under the tobacco control treaty. (Article 19 of the WHO FCTC, Liability). This principle can also be linked with price and tax measures (Article 6 of the WHO FCTC) by incorporating the costs of harms into such measures. The cost can be a fee or added tax per pack of cigarettes, which also could deter smoking. Notably, a few countries have imposed surcharges and fees consistent with the ‘polluter pays’ principle. Policies that make the TI pay for clean-up costs are under consideration in France, the UK, the European Union and the USA (refer to table 1).

The environmental and economic costs of commercial cigarette production estimated here may not account for the full impact of waste from tobacco products, which is probably higher. However, these estimates can be a useful starting point for discussions on assigning accountability to the TI for these costs. However, further studies must take into account the toxic nature of cigarette butt waste and its actual impact on marine, terrestrial and human life. These costs likely will accumulate, just as do the toxic plastic wastes produced by commercial cigarettes.

## supplementary material

10.1136/tc-2022-057795 online supplemental file 1

## Data Availability

Data are available in a public, open access repository. All data relevant to the study are included in the article or uploaded as supplementary information.
