# Supplementary material for: Tobacco industry accountability for marine pollution: country and global estimates
Source: Tob Control. 2023 Nov 28;33(e2):e057795. doi: 10.1136/tc-2022-057795 (PMC11671907; doi:10.1136/tc-2022-057795)
Supplement: online supplemental file 1 [file tc-33-e2-s001.pdf]

Supplementary Tables

Table 1: Results

The following table presents results calculated for 194 countries using the formula presented in this study.

	Country	Cigarette Consumption (in million tons)	Collection Efficiency Assigned (%)[1]	Waste Management (USD)[1,2]	Loss of Ecosystem Services Lifetime (Marine Pollution) (USD)[3]	Total Costs (USD)
1	Afghanistan	5,894	43	876,272	5,345,715	6,221,986
2	Albania	5,902	60	545,739	5,353,061	5,898,800
3	Algeria	30,062	68	3,321,218	109,058,709	112,379,927
4	Andorra	434	100	66,274	393,349	459,623
5	Angola	4,611	23.1	892,104	16,727,250	17,619,354
6	Antigua and Barbuda	6	98.61	961	28,569	29,530
7	Argentina	38,648	89.91	2,883,616	175,261,005	178,144,620
8	Armenia	4,894	85	379,494	4,438,664	4,818,158
9	Australia	18,096	98	2,726,238	32,258,789	34,985,027
10	Austria	18,096	97.77	2,765,383	49,519,293	52,284,676
11	Azerbaijan	11,662	56	1,106,181	10,577,243	11,683,424
12	Bahamas	136	98	20,777	618,547	639,323
13	Bahrain	1,210	98	184,293	15,362,637	15,546,930
14	Bangladesh	86,149	52	11,538,288	1,018,482,432	1,030,020,720
15	Barbados	64	90	9,608	289,774	299,382

16	Belarus	23,303	93.2	1,692,940	21,134,986	22,827,926
17	Belgium	23,052	97.74	3,477,843	61,820,911	65,298,754
18	Belize	42	85.2	3,313	190,915	194,228
19	Benin	1,324	68	146,254	4,802,536	4,948,790
20	Bhutan	927	68	102,415	11,770,531	11,872,946
21	Bolivia	2,158	57.6	279,892	9,785,648	10,065,540
22	Bosnia and Herzegovina	5,822	73.99	489,686	5,280,051	5,769,737
23	Botswana	680	85	52,721	2,466,569	2,519,291
24	Brazil	70,700	90.81	5,256,193	322,451,803	327,707,996
25	Brunei Darussalam	3	98	487	40,632	41,119
26	Bulgaria	11,073	98.3	756,112	9,742,581	10,498,693
27	Burkina Faso	4,156	34.52	688,274	15,032,564	15,720,838
28	Burundi	1,521	43	226,126	5,517,946	5,744,072
29	Cabo Verde	148	70.6	15,629	536,558	552,187
30	Cambodia	7,885	68	871,105	100,115,536	100,986,641
31	Cameroon	2,360	61.6	288,594	8,559,891	8,848,485
32	Canada	31,403	99	4,790,958	85,442,461	90,233,418
33	Central African Republic	652	43	96,888	2,364,264	2,461,152

34	Chad	2,124	43	315,729	7,704,446	8,020,175
35	Chile	11,177	95	1,700,449	50,977,030	52,677,480
36	China	2,350,500	94	153,568,981	3,626,958,059	3,780,527,041
37	Colombia	6,640	80.6	507,302	27,880,216	28,387,519
38	Comoros	283	68	31,266	1,026,679	1,057,945
39	Congo (Democratic Republic of the)	5,525	43	821,353	20,042,734	20,864,087
40	Congo (Republic of the)	531	68	58,709	1,927,835	1,986,544
41	Cook Islands	13	85	1,029	168,513	169,542
42	Costa Rica	1,560	90.4	115,917	7,072,930	7,188,846
43	Côte d'Ivoire	4,730	68	522,572	17,159,688	17,682,261
44	Croatia	5,685	97.76	865,593	5,155,978	6,021,572
45	Cuba	2,233	76.9	150,780	7,247,750	7,398,530
46	Cyprus	1,509	92.42	227,771	1,368,512	1,596,283
47	Czech Republic	21,855	100	3,303,192	77,920,383	81,223,575
48	Denmark	6,193	100	896,410	15,447,911	16,344,321
49	Djibouti	477	68	52,710	1,730,843	1,783,553
50	Dominica	8	94	599	37,639	38,238
51	Dominican Republic	1,640	85	127,193	7,438,435	7,565,628

52	Ecuador	1,092	85	84,677	4,952,003	5,036,680
53	Egypt	90,009	68	9,730,462	315,473,205	325,203,666
54	El Salvador	935	78.8	84,587	4,237,772	4,322,359
55	Equatorial Guinea	174	85	13,461	629,793	643,255
56	Eritrea	407	85	31,568	1,476,894	1,508,462
57	Estonia	1,951	86.89	291,904	5,309,255	5,601,159
58	Eswatini	0.0057013	68	1	21	21
59	Ethiopia	6,942	50	933,163	25,184,473	26,117,636
60	Fiji	315	85	24,395	3,994,616	4,019,011
61	Finland	5,070	100	774,678	13,793,505	14,568,183
62	France	57,743	100	8,466,962	147,096,198	155,563,160
63	Gabon	501	23.6	57,231	1,818,274	1,875,505
64	Gambia	276	43	40,973	999,833	1,040,806
65	Georgia	6,287	60	581,273	5,701,606	6,282,879
66	Germany	114,437	97.55	17,272,500	307,255,882	324,528,382
67	Ghana	696	68	76,850	2,523,526	2,600,376
68	Greece	19,384	98	2,948,237	52,619,577	55,567,814
69	Grenada	13	85	969	56,685	57,654
70	Guatemala	1,189	77.7	97,403	5,393,239	5,490,643

71	Guinea	2,357	43	350,473	8,552,273	8,902,746
72	Guinea-Bissau	28	43	4,207	102,668	106,875
73	Guyana	199	89	14,941	901,519	916,459
74	Haiti	1,034	68	114,270	4,690,345	4,804,615
75	Honduras	2,654	64.6	309,884	12,034,909	12,344,794
76	Hungary	17,465	99.94	2,652,963	47,082,164	49,735,127
77	Iceland	225	100	34,352	611,654	646,006
78	India	84,861	68	8,231,212	248,631,121	256,862,333
79	Indonesia	316,394	45	43,292,863	3,348,278,461	3,391,571,324
80	Iran (Islamic Republic of)	57,240	100	2,757,112	187,514,134	190,271,246
81	Iraq	26,295	76	2,046,052	86,139,879	88,185,931
82	Ireland	3,539	92.04	533,933	9,630,014	10,163,946
83	Israel	7,566	98	1,152,428	27,447,520	28,599,949
84	Italy	78,064	88.92	11,546,069	207,523,051	219,069,121
85	Jamaica	674	76	55,843	3,054,189	3,110,032
86	Japan	173,999	99.9	26,681,622	1,745,880,761	1,772,562,383
87	Jordan	11,565	85	896,798	41,956,708	42,853,506
88	Kazakhstan	23,379	77	1,924,467	21,203,462	23,127,929
89	Kenya	7,287	40	1,182,312	26,437,529	27,619,841

90	Kiribati	104	54	14,195	1,321,804	1,335,999
91	Korea (Democratic People's Republic of)	19,840	43	2,949,540	251,912,208	254,861,748
92	Korea (Republic of)	72,628	99.9	11,096,642	724,579,819	735,676,461
93	Kuwait	4,384	100	669,941	15,904,818	16,574,759
94	Kyrgyzstan	2,135	68	235,909	1,936,632	2,172,541
95	Lao PDR	5,064	55	681,142	64,297,415	64,978,558
96	Latvia	1,992	84.36	296,786	5,420,539	5,717,324
97	Lebanon	9,330	85	723,435	33,845,944	34,569,379
98	Lesotho	620	20	123,432	2,247,810	2,371,242
99	Liberia	413	43	61,445	1,499,387	1,560,832
100	Libya	1,458	85	113,088	5,290,843	5,403,932
101	Lithuania	3,210	98.69	489,447	8,733,211	9,222,659
102	Luxembourg	3,079	100	3,079	8,376,776	8,379,855
103	Madagascar	5,897	17.7	1,180,385	21,394,105	22,574,490
104	Malawi	2,357	43	350,458	8,551,910	8,902,368
105	Malaysia	10,283	95	727,122	128,088,220	128,815,342
106	Maldives	151	38.2	15,874	1,910,965	1,926,839

107	Mali	3,021	43	449,071	10,958,257	11,407,327
108	Malta	550	96.65	83,625	499,010	582,634
109	Marshall Islands	3	49	253	32,445	32,698
110	Mauritania	76	68	8,385	275,353	283,739
111	Mauritius	562	83.7315068	44,036	2,040,298	2,084,334
112	Mexico	30,632	93.4	2,091,423	126,067,197	128,158,620
113	Micronesia (Federated States of)	41	8	9,139	524,130	533,269
114	Moldova (Republic of)	5,982	85	463,853	5,425,346	5,889,199
115	Mongolia	4,256	68	470,194	54,039,052	54,509,245
116	Montenegro	562	96.99	39,578	509,984	549,562
117	Morocco	17,302	68	1,612,015	47,266,166	48,878,181
118	Mozambique	7,916	52.5	1,023,788	28,717,265	29,741,053
119	Myanmar	8,989	60	1,126,031	114,136,054	115,262,084
120	Namibia	476	85	36,887	1,725,764	1,762,651
121	Nauru	21	100	3,270	271,679	274,949
122	Nepal	10,069	62.3	1,218,463	127,844,214	129,062,677
123	The Netherlands	20,901	100	3,087,975	53,896,176	56,984,152
124	New Zealand	2,508	97	381,420	4,549,494	4,930,914

125	Nicaragua	1,421	92.3	93,167	6,444,406	6,537,574
126	Niger	1,217	43	180,975	4,416,171	4,597,146
127	Nigeria	17,029	68	1,689,464	51,844,464	53,533,927
128	Niue	1	85	74	12,184	12,259
129	North Macedonia	4,819	76.61	397,785	4,370,370	4,768,154
130	Norway	2,392	99	364,938	8,677,796	9,042,734
131	Oman	996	98	151,696	3,612,967	3,764,663
132	Pakistan	45,600	68	5,037,908	165,429,555	170,467,463
133	Palau	51	77	7,566	652,820	660,387
134	Occupied Palestinian territory	2,051	43	304,900	7,440,207	7,745,107
135	Panama	640	84.9	49,650	2,901,366	2,951,016
136	Papua New Guinea	8,306	60	1,040,510	105,467,509	106,508,018
137	Paraguay	1,817	46.6	182,578	8,241,548	8,424,126
138	Peru	2,246	82.93	176,912	10,183,804	10,360,716
139	Philippines	79,074	68	8,656,535	989,619,096	998,275,631
140	Poland	44,872	100	6,329,757	107,294,841	113,624,598
141	Portugal	10,207	100	1,554,003	27,611,447	29,165,450
142	Qatar	1,973	100	301,451	7,156,642	7,458,093

143	Romania	19,746	87.45	1,239,550	12,971,675	14,211,226
144	Russian Federation	278,472	85	21,593,495	252,563,134	274,156,629
145	Rwanda	664	43	98,672	2,407,798	2,506,470
146	Saint Kitts and Nevis	3	95	406	12,152	12,558
147	Saint Lucia	80	100	5,467	361,424	366,890
148	Saint Vincent and the Grenadines	22	85	1,675	97,952	99,627
149	Samoa	181	100	9,264	2,291,889	2,301,153
150	San Marino	140	98	21,261	126,594	147,855
151	Sao Tome and Principe	18,856	48.4	2,766,382	68,405,860	71,172,242
152	Saudi Arabia	30,912	100	4,433,028	101,265,495	105,698,523
153	Senegal	3,150	21.4	619,444	11,429,151	12,048,595
154	Serbia	14,028	74.67	1,174,215	12,722,385	13,896,601
155	Seychelles	40	98	6,139	146,202	152,341
156	Sierra Leone	1,789	43	266,029	6,491,659	6,757,688
157	Singapore	2,828	100	432,151	35,908,371	36,340,522
158	Slovakia	6,944	94.34	1,051,494	18,894,069	19,945,563
159	Slovenia	3,937	93.84	595,592	10,710,747	11,306,340
160	Solomon Islands	514	12	109,975	6,525,216	6,635,191

161	Somalia	1,175	43	174,746	4,264,164	4,438,910
162	South Africa	19,778	85	1,500,674	69,320,134	70,820,808
163	South Sudan	2,839	43	422,057	10,299,078	10,721,135
164	Spain	59,071	100	8,993,856	159,802,339	168,796,195
165	Sri Lanka	4,013	68	443,303	50,948,494	51,391,796
166	Sudan	8,371	43	1,244,526	30,369,021	31,613,546
167	Suriname	206	48.7	20,437	934,169	954,606
168	Sweden	5,860	100	888,905	15,760,410	16,649,315
169	Switzerland	10,651	99	1,632,602	38,926,804	40,559,406
170	Syrian Arab Republic	27,197	74	2,327,489	98,667,846	100,995,335
171	Tajikistan	2,035	38.25	336,726	1,845,573	2,182,300
172	Tanzania	5,500	68	607,642	19,953,126	20,560,769
173	Thailand	47,122	85	3,640,619	594,886,291	598,526,910
174	Timor-Leste	829	68	91,589	10,526,306	10,617,896
175	Togo	640	43	95,148	2,321,818	2,416,967
176	Tonga	67	71	5,772	853,268	859,041
177	Trinidad and Tobago	738	94.31	111,805	3,348,497	3,460,302
178	Tunisia	13,750	65	1,595,360	49,882,815	51,478,175

179	Turkey	105,763	88.12	7,802,960	368,484,900	376,287,861
180	Turkmenistan	3,763	85	291,755	3,412,438	3,704,193
181	Tuvalu	4	47	392	49,711	50,104
182	Uganda	4,116	37.5	657,980	14,931,831	15,589,811
183	Ukraine	71,947	75.86	6,903,369	65,253,162	72,156,531
184	United Arab Emirates	5,960	98	907,832	21,621,933	22,529,765
185	United Kingdom	44,875	97.43	6,853,922	163,732,542	170,586,464
186	United States of America	266,341	100	40,418,271	716,775,082	757,193,353
187	Uruguay	1,824	98	277,833	8,271,478	8,549,311
188	Uzbekistan	10,737	68	1,186,239	9,738,123	10,924,363
189	Vanuatu	185	12	39,526	2,345,218	2,384,744
190	Venezuela	9,014	100	618,272	40,876,700	41,494,972
191	Vietnam	76,251	72	7,860,420	968,192,796	976,053,216
192	Yemen	6,999	20	1,368,199	25,391,986	26,760,184
193	Zambia	1,320	20	262,880	4,787,299	5,050,179
194	Zimbabwe	1,147	68	126,666	4,159,320	4,285,986
	TOTAL	-	-	547,644,658	20,408,613,934	20,956,258,592

Table 2: Countries with Policies on Plastics/Single-Use Plastics (SUPs)

The following table lists the countries with different policies on plastics or single-use plastics.

S. No.	Plastics Policy	Countries[4]
1.	**Countries that expressed commitment to a legally binding United Nations (UN)Treaty on plastic pollution**	175 UN Member States[5]
2.	**Countries/groups of countries with a ban on SUPs**[i]	Antigua and Barbuda, Argentina, Australia, Bangladesh, Belgium, Belize, Benin, Bhutan, Botswana, Brazil, Bulgaria, Burkina Faso, Cameroon, Canada, Cape Verde, Chad, Chile, China, Colombia, Croatia, Cyprus, Czech Republic, Côte d'Ivoire, Denmark, Ecuador, Egypt, Eritrea, Estonia, Ethiopia, European Union, Fiji, France, Gambia, Greece, Guatemala, Guinea-Bissau, Guyana, Haiti, Honduras, Hungary, India, Indonesia, Ireland, Israel, Italy, Kenya, Latvia, Lithuania, Malawi, Malaysia, Mali, Malta, Marshall Islands, Mauritania, Mauritius, Mexico, Mongolia, Morocco, Mozambique, Myanmar, Netherlands, Niger, Pakistan, Palau, Panama, Papua NewGuinea, Philippines, Portugal, Romania, Rwanda, Senegal, Slovakia,Somalia, South Africa, Spain, Sri Lanka, Saint Vincent and the Grenadines, Sweden, Tanzania, Tunisia, Uganda, United Kingdom, United States of America, Vanuatu, Vietnam and Zimbabwe
3.	**Countries with a ban on SUPs at the manufacturer or retailer level**[ii]	Antigua and Barbuda, Bangladesh, Benin, Bhutan, Botswana, Burkina Faso, Cameroon, Cape Verde, Chile, China, Colombia, Côted'Ivoire, Croatia, Cyprus, Czech Republic, Eritrea, Estonia, Ethiopia, Fiji, France, Gambia, Greece, Guinea Bissau, Haiti, Ireland, Israel, Italy, Kenya, Lithuania, Malawi, Mali, Marshall Islands, Mauritania, Mauritius, Mongolia, Morocco, Mozambique, Netherlands, Niger, Palau, Panama, Papua New Guinea, Portugal, Romania, Rwanda, Senegal, Slovakia, South Africa, Sri Lanka, Tanzania, Tunisia, Uganda, Vanuatu and Zimbabwe

[i] This includes countries with a ban on SUPs at any level- city-wide or state-wide or federal/ national or sub-national levels.
[ii] This includes countries with a ban on SUPs at the national level.

References

[1] The World Bank Group. What a Waste 2.0: A Global Snapshot of Solid Waste Management to 2050. https://datatopics.worldbank.org/what-a-waste/trends_in_solid_waste_management.html
[2] OECD. Global Plastics Outlook: Economic Drivers, Environmental Impacts and Policy Options, 2022. https://www.oecd-ilibrary.org/sites/c2744069-en/index.html?itemId=%2Fcontent%2Fcomponent%2Fc2744069-en
[3] WWF & Dalberg. Plastics: The cost to society, the environment and the economy, 2021. https://media.wwf.no/assets/attachments/Plastics-the-cost-to-society-the-environment-and-the-economy-WWF-report.pdf
[4] United Nations Environment Programme. Legal Limits on Single-Use Plastics and Microplastics: A Global Review of National Laws and Regulations, December 2018. https://www.unep.org/resources/publication/legal-limits-single-use-plastics-and-microplastics-global-review-national
[5] United Nations News [website]. Nations sign up to end global scourge of plastic pollution, March 2022. https://news.un.org/en/story/2022/03/1113142
